# Herbal Extracts Mixed with Essential Oils: A Network Approach for Gastric and Intestinal Motility Disorders

**DOI:** 10.3390/nu16244357

**Published:** 2024-12-17

**Authors:** Roberta Budriesi, Ivan Corazza, Simone Roncioni, Roberta Scanferlato, Dalila De Luca, Carla Marzetti, Roberto Gotti, Nicola Rizzardi, Christian Bergamini, Matteo Micucci, Davide Roncarati, Laura Beatrice Mattioli

**Affiliations:** 1 Food Chemistry and Nutraceutical Laboratory, Department of Pharmacy and Biotechnology (FaBiT), Alma Mater Studiorum-University of Bologna, Via Belmeloro 6, 40126 Bologna, Italy; laurabeatrice.mattioli@unibo.it; 2 Department of Medical and Surgical Sciences (DIMEC), Alma Mater Studiorum-University of Bologna, 40138 Bologna, Italy; ivan.corazza@unibo.it; 3 Valsambro S.r.l., 40121 Bologna, Italy; simone.roncioni@gmail.com (S.R.); r.scanferlato@hotmail.com (R.S.); dalila.deluca@valsambro.it (D.D.L.); carla.marzetti@valsambro.it (C.M.); 4 Department of Pharmacy and Biotechnology (FaBiT), University of Bologna, Via Belmeloro 6, 40126 Bologna, Italy; 5 Department of Pharmacy and Biotechnology (FaBiT), University of Bologna, Via Irnerio 48, 40126 Bologna, Italy; nicola.rizzardi2@unibo.it (N.R.); christian.bergamini2@unibo.it (C.B.); 6 Department of Biomolecular Sciences, University of Urbino “Carlo Bo”, 61029 Urbino, Italy; matteo.micucci@uniurb.it; 7 Department of Pharmacy and Biotechnology (FaBiT), University of Bologna, Via Selmi 3, 40126 Bologna, Italy; davide.roncarati@unibo.it

**Keywords:** *Asparagus racemosus* Willd., *Tabebuia avellanedae* Lorentz, *Glycyrrhiza glabra* L., *Helicobacter pylori*, tissues contractility, antioxidant activity, essential oils

## Abstract

Background: Three herbal extracts (*Asparagus racemosus* Willd., *Tabebuia avellanedae* Lorentz, and *Glycyrrhiza glabra* L.) were mixed with three essential oils (*Foeniculum vulgare* Mill., *Mentha piperita* L., and *Pimpinella anisum* L.) to formulate a product (HEMEO) whose active compounds include saponins and steroids in *Asparagus racemosus*, known for their anti-inflammatory properties; glycyrrhizin and flavonoids in *Glycyrrhiza glabra*, which exhibit gastroprotective and antispasmodic effects; menthol in *Mentha piperita*, contributing with antispasmodic and antimicrobial properties; and anethole and polyphenols in *Pimpinella anisum*, which modulate intestinal motility and offer antimicrobial activity. Objective: HEMEO was formulated for applications in intestinal motility disorders. Methods: HEMEO was evaluated for spontaneous and induced motility effects in isolated guinea pig ileum, colon, and stomach. Ex vivo experiments were conducted using LabChart software v7.0, and the product’s antibacterial action against *Helicobacter pylori* and its antioxidant effects were assessed through disc diffusion and FRAP assays. The presence of the volatile compounds in the formulation was confirmed by GC-MS analysis; the TPC of HEMEO, determined using the Folin–Ciocalteu method, was 9.925 ± 0.42 mg GAE/g. Conclusions: HEMEO showed a phenolic content correlated with its antioxidant potential and in addition inhibited *H. pylori* growth and demonstrated notable antioxidant properties, suggesting its role as a supportive agent in digestive processes and in managing motility disorders.

## 1. Introduction

The gastrointestinal (GI) tract performs various functions, including digestion and absorption [1]. Motility, the muscular activity of the GI tract, plays a key role in the digestive process across different parts of the intestinal tract [2,3]. Normal motility involves a sequence of muscular contractions from top to bottom [4]. When motility is abnormal, with contractions that are too rapid, slow, or disorganized, pain can arise due to brain–gut communication [5]. Central nervous system processing in visceral hypersensitivity causes functional dysfunctions and pain [6,7]. “Intestinal motility disorders” are characterized by abnormal intestinal contractions (spasms and paralysis), where the gut loses its ability to coordinate motor function due to endogenous or exogenous causes or infection. With its muscular solid walls, the stomach holds food and mixes it with acid and enzymes into a liquid paste, playing a crucial role in the digestive process. However, food or drugs can compromise the delicate balance that regulates this organ’s functionality [8]. Functional minor and colonic disorders involve middle or lower GI tract symptoms, impacting the absorption of nutrients, vitamins (small intestine), water, and electrolytes (colon). Functional motility disorders include inflammatory bowel syndrome (IBS), functional bloating, functional constipation, functional diarrhea, and unspecified functional bowel disorders. The human gut microbiome, a heterogeneous microbial system, regulates intestinal physiology and pathology. Disruption of this microbial equilibrium can break the epithelial barrier, causing hypersensitivity and abnormalities in gut motility [9].

This paper focuses on a pathogenic bacterium, *Helicobacter pylori* (*H. pylori*), which infects and causes inflammation and ulcers in the stomach or small intestine. *H. pylori* is very common, affecting about two-thirds of the world’s population [10]. Many studies demonstrate an association between H. pylori and disorders outside of the stomach [11], demonstrating how the infection also influences pathologies of the extragastric intestinal tract [12]. In particular, *H. pylori* infection appears to be able to modify the intestinal microbiota by altering the release of local chemical mediators that influence the host’s immune response [13].

Herbal mixtures are widely used in traditional Chinese medicine to target various aspects of pathologies [14,15,16,17,18]. In this context, a multitarget approach to addressing different mechanisms associated with the same pathology is of great importance for effective symptom management. It is well-established that disorders related to impaired motility often require various drugs acting on multiple therapeutic targets [19]. The “network target” approach we propose is based on a mixture of organic compounds within the extracts, capable of interacting with multiple targets [20]. This strategy involves evaluating the activity of the extracts on individual targets associated with the pathology. Furthermore, combining different herbal extracts aims to broaden the range of targets the therapy addresses while minimizing effects on targets unrelated to the pathological network. HEMEO, the focus of this study, is a multi-component herbal formula designed to address GI motility disorders by leveraging the active compounds in each of its herbal and essential oil constituents. Each plant extract contributes specific bioactive compounds with targeted effects. The herbal mixture consists of extracts from *Asparagus racemosus* Willd. Oberm (racemose asparagus), *Tabebuia avellanedae* Lorentz ex Griseb (lapacho), and *Glycyrrhiza glabra* L. (licorice). *Asparagus racemosus* contains saponins and steroidal compounds, primarily shatavarins, which exhibit anti-inflammatory and antiulcerogenic impact, making this herb useful for managing gastric discomfort and supporting digestive health [21,22,23,24]. Studies by Bhatnagar et al. demonstrate that *Asparagus racemosus* reduces gastric emptying time [25], a phenomenon linked to duodenal ulcer and gastric HCl release [26], and presents significant antidiarrheal action by inhibiting intestinal motility [27]. *Tabebuia avellanedae,* known as “lapacho”, has gastroprotective action [28] and is used in traditional medicine for treating ulcers and infections [29]. *Glycyrrhiza glabra* roots are rich in glycyrrhizin (18 β-glycyrrhetinic acid), an essential compound for preventing gastroduodenal ulcers and reducing pain. This gastroprotective action is complemented by flavonoids, which collectively contribute to licorice’s role in soothing the GI tract. However, licorice use has been associated with side effects, particularly hypertension, due to glycyrrhizin’s influence on sodium retention and potassium loss [30]. The gastrointestinal effects are further enhanced by isoliquiritigenin, a bioactive flavonoid that induces spasmolytic effects by blocking calcium channels, helping to alleviate smooth muscle spasms and thus improving GI comfort [31]. The distinctive feature of the selected herbal formulation lies in the presence of essential oils. Essential oils, in general, inhibit critical steps of bacterial infections, including biofilm formation and toxin production, which are pivotal for maintaining intestinal homeostasis [32]. Moreover, the intestinal microbiota plays a crucial role in maintaining colon homeostasis, mitigating many adverse effects caused by chemical compounds in foods and during infections. In this context, the selective activity of essential oils against pathogenic bacterial populations presents a particularly compelling area of interest [33].

Three essential oils from *Foeniculum Vulgare* Mill. (fennel), *Mentha piperita* L. (peppermint), and *Pimpinella Anisum* L. (anise) complement the herbal extract. Fennel is commonly used in traditional medicine for various digestive disorders [34] and is praised for its antimicrobial and antifungal action [35]. Its essential oil regulates intestinal motility and is used for gastrointestinal atony and stomach heaviness [36,37,38]. Anethole, found mainly in fennel seeds, has anti-cancer properties [39]. Peppermint, rich in menthol, this provides as an essential oil antispasmodic and antimicrobial properties. Menthol interacts with calcium channels and provides soothing effects on intestinal muscle, making it beneficial in managing spasms related to GI disturbances [40,41]. Both fennel and peppermint have antispasmodic actions on smooth muscles and influence gastric and salivary secretion. Anise essential oil contains anethole and polyphenols, which modulate GI motility and offer antimicrobial effects that may help balance intestinal flora. Anise is widely used to alleviate bloating and discomfort associated with digestive issues [38,42].

All these extracts and essential oils act on the gastrointestinal tract and modulate targets linked to GI functional disorders. Therapies based on the “one drug, one target, one disease” approach have gradually shifted towards adopting multiple active component treatment strategies [43]. Our study analyzed the synergistic effect of active ingredients on a network of gastrointestinal targets. Therefore, it is a multitarget-get-multicomponent approach, which allows for the pathology to be treated globally, considering “more drugs and more targets”.

**Figure 1:**
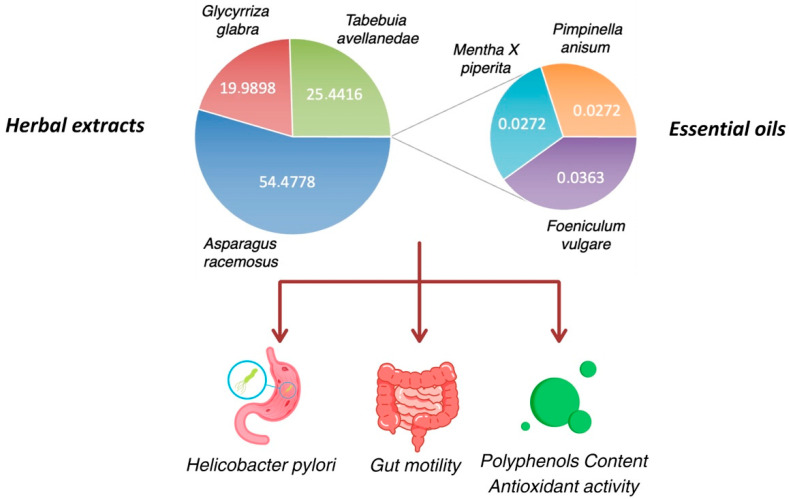
Overview of experimental design of HEMEO.

This study evaluated the effect of HEMEO on the basal and induced contractility of guinea pigs’ stomach, ileum, and colon, as well as its effect on *H. pylori* and its antioxidant properties. The cardiovascular impact of HEMEO was also studied to provide a comprehensive overview of this herbal mixture’s off-target activity. Figure 1 shows the experimental design.

## 2. Materials and Methods

### 2.1. HEMEO Chemical Composition

**Figure 2:**
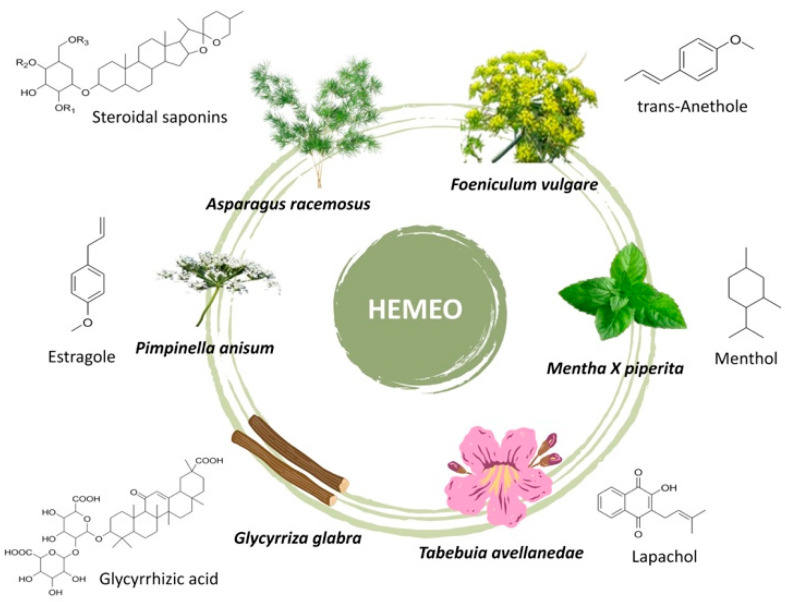
Plants pictures and chemical structure of key compounds found in the plant extracts and essential oils.

The composition of the herbal extracts mixed with essential oils, kindly provided by Homeo Sapiens, Cesena (FC), Italy, is as follows: *Asparagus racemosus* Willd. Oberm 300 mg (54.4778%); *Tabebuia avellanedae* Lorentz ex Griseb 140 mg (25.4416%), containing 3% naphthoquinones as lapachol equivalent to 4.2 mg; *Glycyrrhiza glabra* L. 110 mg (19.9898%), containing triterpenic saponins with 19% glycyrrhizic acid; *Foeniculum vulgare* Mill. essential oil 0.200 mg (0.0363%); *Mentha X piperita* L. essential oil 0.150 mg (0.0272%); *Pimpinella anisum* L. essential oil 0.150 mg (0.0272%) (Figure 2).

#### 2.1.1. Identification of Essential Oils Components

GC-MS instrumentation and conditions. The chromatographic experiments to identify the terpenes associated with the essential oils of HEMEO were carried out on a GC-MS Gas Chromatograph by Agilent Technologies (Santa Clara, CA, USA), model 8890 combined with the Mass detector (MS) Agilent model 5977B. The chromatographic conditions were as follows: the capillary column was an Rtx^®^ 5 MS column (30 m × 0.25 mm ID, 0.25 μm) Crossbond^®^ (5% phenyl–95% dimethylpolysiloxane) by (Restek, Cernusco sul Naviglio, Milano, Italy); the carrier gas was helium at the flow rate of 1 mL/min; the temperature program was initial temperature of 40 °C (hold time: 5 min), followed by a ramp at 7 °C/min to 230 °C (hold time: 5 min). The injector base, transfer line, and ionization source temperatures were 280, 250, and 200 °C, respectively. The Gas Chromatograph (GC) was operated in split mode using a split ratio of 1:50. The electron ionization (EI at 70 eV) was used, and the mass spectra were recorded in full scan mode (50–650 amu) to collect the total ion current (TIC) chromatograms. The terpenes were identified by comparing retention time and mass spectrum with authentic standards as reference. The identity confirmation was carried out by comparing the mass spectra of the analytes with those of the NIST (National Institute of Standards and Technology, Gaithersburg, MD, USA) library version 2.4 (2020).

Sample preparation for GC-MS analysis. The samples to be subjected to GC-MS analysis were prepared by extracting an amount of 1 mg of the HEMEO powder using dichloromethane (1 mL) under ultrasonication using an ultrasonic bath for 5 min. The suspension was filtered (0.45 µm) before injecting into the gas chromatograph.

#### 2.1.2. Determination of Total Phenolic Content (TPC)

The total phenolic content (TPC) in HEMEO was determined using a slightly modified Folin–Ciocalteu method [44,45]. A calibration curve was established using a series of gallic acid standards (50–500 µg/mL of gallic acid solution in water). For each standard and sample, 50 µL was combined with 250 µL of Folin–Ciocalteu reagent and allowed to react for 5 min. Subsequently, 1 mL of 20% Na_2_CO_3_ was added, and the mixture was diluted with demineralized water to a final volume of 5 mL. The solutions were then incubated at room temperature for 30 min, and absorbance was measured at 760 nm using a spectrophotometer (Jasco V-730, Jasco Europe SrL, Cremella, Italy). The TPC of the samples was expressed as Gallic Acid Equivalents (GAEs) in mg/g, calculated from the calibration curve.

### 2.2. Ex Vivo Studies

#### 2.2.1. Animals

Male guinea pigs (200–400 g) obtained from Charles River (Calco, Como, Italy) were used. The animals were housed according to Directive 2010/63/EU of the European Parliament and the Council and the WMA Statement on Animal Use in Biomedical Research. All procedures followed the guidelines of the animal care and use committee of the University of Bologna (Bologna, Italy) (“Protocol 2DBFE.N.YEV” by the Comitato Etico Scientifico for Animal Research Protocols according to D.L. vo 116/92 and approved by the Ministry of Health in December 2023). The animals were sacrificed by cervical dislocation, and the organs required were set up rapidly under a suitable resting tension in a 15 mL organ bath containing appropriate physiological salt solution (PSS) warmed and buffered to pH 7.4 by saturation with 95% O_2_–5% CO_2_ gas and used as described below.

Guinea pig gastric fundus. As previously described [46], the stomach was removed, opened along the mesentery of the greater curvature, and rinsed with Krebs bicarbonate-buffered solution maintained at 37 °C. Fundus muscle strips were cut parallel to the circular muscle layer, tied at each end with sutures, and hung in the organ baths containing Krebs bicarbonate-buffered solution.

Guinea pig ileum. The terminal portion of the ileum (3–4 cm near the ileo–caecal junction) was cleaned, and segments 2–3 cm long of ileum were set up under 1 g tension at 37 °C in organ baths containing Tyrode solution of the following composition (mM): NaCl, 118; KCl, 4.75; CaCl_2_, 2.54; MgSO_4_·7H_2_O, 1.20; KH_2_PO_4_·2H_2_O, 1.19; NaHCO_3_ 25; and glucose 11. The two segments obtained (2–3 cm) were set up under 1 g tension in the longitudinal direction along the intestinal wall. Tissues were allowed to equilibrate for at least 30 min, during which the bathing solution was changed every 10 min.

Guinea pig proximal colon. Starting approximately 1 cm distal from the caecocolonic junction, two segments of about 1 cm of the guinea pig’s proximal colon were cut. The proximal colon was cleaned by rinsing with De Jalon solution of the following composition (mM): NaCl, 155; KCl, 5.6; CaCl_2_, 0.5; NaHCO_3_, 6.0; and glucose, 2.8; and the mesenteric tissue was removed. The two segments were suspended in organ baths containing gassed, warm De Jalon solution under a load of 1 g maintained at 37 °C. Tension changes in longitudinal muscle length were recorded. Tissues were allowed to equilibrate for at least 30 min, during which the bathing solution was changed every 10 min.

Guinea pig heart. After thoracotomy, the heart was immediately removed and washed by perfusion through the aorta with oxygenated Tyrode solution of the following composition (mM): 136.9 NaCl, 5.4 KCl, 2.5 CaCl_2_, 1.0 MgCl_2_, 0.4 NaH_2_PO_4_xH_2_O, 11.9 NaHCO_3_, and 5.5 glucose. The physiological salt solution (PSS) was buffered at pH 7.4 by saturation with 95% O_2_–5% CO_2_ gas, and the temperature was maintained at 35 °C. The following isolated guinea pig heart preparations were used: spontaneously beating right atria and left atria driven at 1 Hz. For each preparation, the left and right atria were dissected from the ventricles, cleaned of excess tissue, and hung vertically in a 15 mL organ bath containing the described PSS. The contractile activity was recorded isometrically using a force transducer (FT 0.3, Grass Instruments Corporation, Quincy, MA, USA) using Power Lab^®^ 8/30 software (AD-Instruments Pty Ltd., Castle Hill, Australia). The left atria were stimulated by rectangular pulses of 0.6–0.8 ms duration and about 50% threshold voltage through two platinum contact electrodes in the lower holding clamp (Grass S88 Stimulator).

The right atria were in spontaneous activity. After the tissues were beaten for several minutes, a length-tension curve was determined, and the muscle length was maintained at that, which elicited 90% of the maximum contractile force observed at the optimal length. A 45–60 min stabilization period was allowed before various agents challenged the atria. During the equilibration period, the bathing solution was changed every 15 min. The method to test inotropy and chronotropic activities was previously described [47].

Guinea pig aortic strips. The aortic strips were prepared as previously described [48].

#### 2.2.2. Spontaneous Contractility on the Ileum and Colon

As previously described [44], the tracing graphs of spontaneous contractions of the gastric fundus, ileum, and proximal colon were continuously recorded with the LabChart 7.0 Pro Software (ADInstruments, Bella Vista, NSW, Australia). After the equilibration period (about 30 to 45 min according to each tissue), cumulative concentration curves (0.1, 0.5, 1, 5, and 10 mg/mL) to HEMEO were constructed. At the end of each single dose, the following parameters were evaluated considering a 5 min stationary period:

- Mean contraction amplitude (MCA), evaluated as the mean force value (g);
- Standard deviations of the force values over the period as an index of the spontaneous contraction variability (SCV);
- Basal spontaneous motor activity (BSMA), as the percentage (%) variation in each mean force value (g) for the control period;
- Spontaneous contractions were investigated in the frequency domain through a standard FFT analysis and a subsequent power spectral density (PSD) plot. The absolute powers of the following frequency bands of interest: low [0.0, 0.2] Hz (LF), medium [0.2, 0.6] Hz (MF), and high [0.6, 1.0] Hz (HF) [49], were then calculated.

The Lab Chart 7 Pro Software performed all the calculations in a post-processing phase. A skilled operator chose the analysis period to avoid errors due to artifacts.

#### 2.2.3. Induced Contractility

Intestinal histamine receptor. As previously described, the H_1_-receptor non-competitive antagonism activity of HEMEO was tested using guinea pig ileum [49]. Briefly, after a stabilization period, concentration–response curves were constructed by cumulative addition of the agonist (histamine). The agonist (histamine) concentration in the organ bath was added only after the response to the previous addition had attained a maximal level and remained steady. Contractions were recorded using a displacement transducer (FT. 03, Grass Instruments, Quincy, MA, USA) using Power Lab 7 Pro software (ADInstruments Pty Ltd., Castle Hill, Australia). In all cases, parallel experiments in which tissues received no antagonist were run to check any sensitivity variation. Concentration–response curves to agonist were obtained at 30 min intervals; the first was discarded, and the second was taken as control. Following incubation with the antagonist (HEMEO), a new concentration–response curve was obtained for the agonist. Tension changes were recorded isotonically.

#### 2.2.4. Cardiovascular Guinea Pig Preparations

Heart histamine receptor. The H_2_-receptor antagonism activity of HEMEO was tested using spontaneously beating right atria [50]. As previously described, after a stabilization period (see below), a cumulative dose–response curve to histamine (0.1–50 μM) was constructed. After washing out for 30 min, an incubation period (30 min) was conducted on the antagonist (HEMEO 0.5, 1, 5 mg/mL). Following incubation, a new curve for the agonist was constructed.

Inotropic and chronotropic activities. To test inotropic and chronotropic activities, the left atria were driven at 1 Hz and spontaneously beat the right atria following the previously described method [48]. After a stabilization period, experiments were carried out in both cases using a single concentration of HEMEO (1 mg/mL). Following incubation (30 min), a new curve for the agonist was constructed.

Spasmolytic activity on K^+^ depolarized aortic strips. The thoracic aorta was excised and immersed in Tyrode solution with the following composition (in mM): 118 NaCl, 4.75 KCl, 2.54 CaCl_2_, 1.20 MgSO_4_, 1.19 KH_2_PO_4_, 25 NaHCO_3_, and 11 glucose. The solution was equilibrated with a gas mixture of 95% O_2_ and 5% CO_2_, maintaining a pH of 7.4. After the equilibration period, guinea pig aortic strips were contracted by exposure to a physiological saline solution (PSS) containing 80 mM KCl, achieved through equimolar substitution of K^+^ for Na^+^. Tension changes in smooth muscle K^+^ 80 mM-induced contraction were recorded isometrically as previously reported [48]. The effect of HEMEO was checked by constructing a cumulative concentration–effect curve.

#### 2.2.5. Statistical Analysis

Experiments were performed in duplicate with tissue from the same animal, and mean values were recorded. The data presented are means ± SEM for a series of experiments with four animals for the gastric fundus and ileum and four to six animals for the proximal colon. Normality was assessed with the Shapiro–Wilk test.

Spontaneous contractility: Comparisons between mean spontaneous contraction amplitudes (MCAs) at different concentrations were performed by one-way ANOVA with Bonferroni’s correction after assessing the data normality through a Kolgomorov–Smirnov test. Differences with *p* < 0.05 were considered statistically significant.

Since statistical analysis results on spontaneous motility are certainly accurate but too sensitive compared to the natural physiological behavior of the tissues, we hypothesized a personal classification scale that considers the percentage changes in the measured parameters compared to baseline values. Thus, the PSD% variations for each band of interest to control were estimated. LF, MF, and HF contractions were considered physiologically relevant when higher or lower than 50% variations for the basal values.

LF, MF, and HF percentage variations were classified depending on their value following these criteria: ≤−500%: “---”; [−500%, −300%]: “--”; [−300%,−50%]: “-”; [−50%, +50%]: “=”; [+50%, +300%]: “+”; [+300%, +500%]: “++”; ≥+500%: “+++”.

Similarly, tone variations were categorized as follows: ≤−100%: “------”; [−100%, −80%]: “-----”; [−80%, −60%]: “----”; [−60%, −40%]: “---”; [−40%, −20%]: “--”; [−20%, 0%]: “-”; Not significant: “=”; [0%, +20%]: “+”; [+20%, +40%]: “++”; [+40%, +60%]: “+++”; [60%, +80%]: “++++”; [+80%, +100%]: “+++++”; ≥+100%: “++++++”.

Induced contractility: the spasmolytic activity of samples was expressed as the percent inhibition of calcium-induced contraction on K^+^-depolarized gastric fundus, ileum, colon, and aorta strips (smooth muscle activity). Data were analyzed by Student’s t-test. The potency of all samples defined as IC_50_ was evaluated from log concentration–response curves (Probit analysis by Litchfield and Wilcoxon, *n* = 6–8) in the appropriate pharmacological preparations. All data are presented as mean ± SEM [51,52,53].

Determination of Dissociation Constants. In functional experiments, the antagonism activity to histamine of HEMEO was estimated by determining the concentration of the non-competitive antagonist that inhibited 50% of the maximum response to the agonist. Three different antagonist concentrations were used, each tested at least four times. A pharmacological computer program [51] was used to analyze the data. A *p* value less than 0.05 was considered significant. All the figures were created using GraphPad software v6.0 [52].

### 2.3. Antimicrobial Disc Diffusion Test

The agar disk diffusion method assessed the antimicrobial activity of the tested compounds against *Helicobacter pylori*. All solutions/suspensions were tested in parallel, using *H. pylori* G27 strain. Bacterial cells were recovered from glycerol stocks on Brucella broth agar plates containing 5% fetal calf serum (FCS), added with Dent’s antibiotic supplement. Plates were incubated in a CO_2_-controlled (9% CO_2_) incubator (Thermo Scientific, Waltham, MA, USA) at 37 °C.

*H. pylori* cells were then collected from plates and resuspended in 0.5 mL of liquid Brucella broth with 5% FCS, and the cell density (OD_600_) of bacterial suspension was determined. Then, bacteria were diluted in melted Brucella broth Soft Agar medium (Brucella broth agar plates containing 0.5% agar) to obtain a final OD_600_ of 0.07, and 6.5 mL of this suspension was poured onto a sterile standard Brucella broth agar plate. After that, sterile paper discs (diameter of 5 mm) were deposited onto the agar plates.

For HEMEO, two concentrations were prepared in sterile water and tested (100 mg/mL and 20 mg/mL). Then, 4 µL of the tested compounds was dropped on the disc. The H_2_O vehicle solutions were used as the negative control, while Kanamycin (2.5 mg/mL in H_2_O) was used as the reference antibiotic. The plates were incubated under microaerophilic conditions at 37 °C for 72 h; then, the diameter of the inhibition zone was measured. This experiment was performed in biological triplicates, and the average inhibition diameter was calculated for each tested concentration [54].

### 2.4. Antioxidant Capacity Assay

The FRAP assay evaluated the antioxidant capacity of HEMEO according to Benzie et al. [55]. Briefly, different amounts of compounds were added to the FRAP reagent solution, and the formation of the Fe^2+^–tripyridyltriazine complex was monitored spectrophotometrically at λ 595 nm, T = 30 °C, at time 0 (Abs0), and after 3 min (Abs3) using a Jasco V-750 spectrophotometer equipped with a stirring device and thermostatic control. Standard Fe^2+^ was used for the calibration curve. Data are expressed as ΔAbs (Abs3 − Abs0) on mg. All measurements were performed in triplicate ± SD.

## 3. Results

### 3.1. HEMEO Essential Oils Components Qualitative Profile

To verify the persistence of the three selected essential oils in HEMEO, a GC-MS qualitative analysis of the dichloromethane extracts obtained from the formulated mixture was undertaken. The investigation focused on detecting the most characteristic terpenes of the three essential oils, i.e., trans-anethole, menthol, and estragole (Figures S1–S3, Supplementary Materials). The selected terpenes were unambiguously identified by comparison with the retention time of standard solutions and by their MS spectra using the library (NIST version 2.4) for comparison. In particular, trans-anethole (Figure S1) confirmed the presence of *Pimpinella anisum* and *Foeniculum vulgare* [56,57] as it is the principal and most characteristic terpene in these essential oils; in addition, estragole (Figure S2), a phenylpropanoid typically contained in fennel, was a further hint of the persistence of *Pimpinella anisum* in the formulation [57,58]. Eventually, menthol (Figure S3) was detected, and the presence of *Mentha piperita* essential oil [59] was ascertained. The peak area of the characteristic terpenes was reasonably assumed to be the response to assess the stability of the essential oils. The peak area RSD% (*n* = 9) measured at the time of product preparation and after 3, 6, and 12 months of storage under conventional conditions (closed, room temperature, dark) was <5.2% (*n* = 9).

As expected, several minor components were observed in the chromatograms due to the presence of the other terpenes of the essential oils and the volatile components of the formulation; interestingly, the selected terpenes were the most represented, demonstrating that the adopted formulation allows for embedding the essential oils, ensuring their stability during storage.

### 3.2. Total Phenolic Content (TPC) of HEMEO

The TPC of HEMEO was quantified using the Folin–Ciocalteu assay. As shown in Table 1, the result expressed as mg of gallic acid equivalent (GAE) per g of HEMEO was 9.925 ± 0.42 mg GAE/g HEMEO (*n* = 5).

### 3.3. Ex Vivo Studies

**Figure 3:**
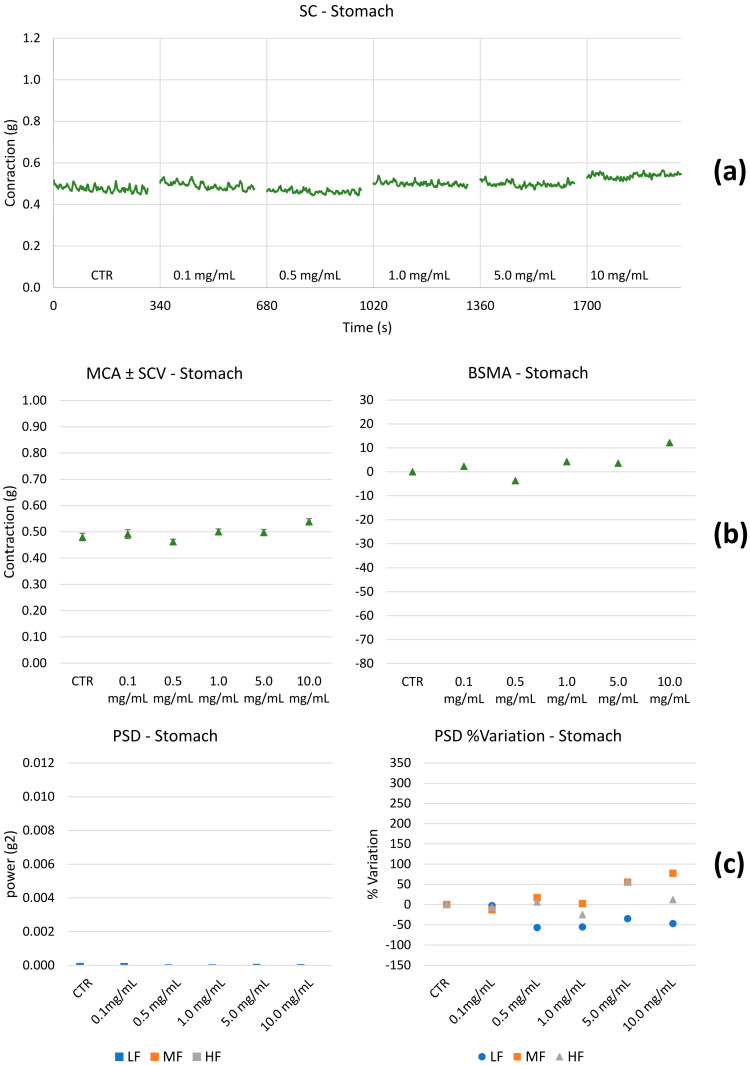
Example of experimental original recording of the concentration–response curve of HEMEO on spontaneous longitudinal (Long) stomach basal contractility. (**a**) Spontaneous contraction (SC) signals for each concentration; (**b**) mean contraction amplitude (MCA) and spontaneous contraction variability (SCV), represented as error bars in the MCA plot and contraction percentage variation for the control (BSMA) for each considered condition; all the comparisons resulted in being statistically significant (*p* < 0.05); (**c**) power spectral density (PSD) and percentage variations. Despite statistically significant differences between muscular tones at different HEMEO concentrations, the MCA variations are not physiologically significant (less than 20%). MF and HF bands values increase over 50% only for concentrations of 5.0 mg/mL and 10 mg/mL, suggesting a slight increase in mixing and pain.

**Figure 4:**
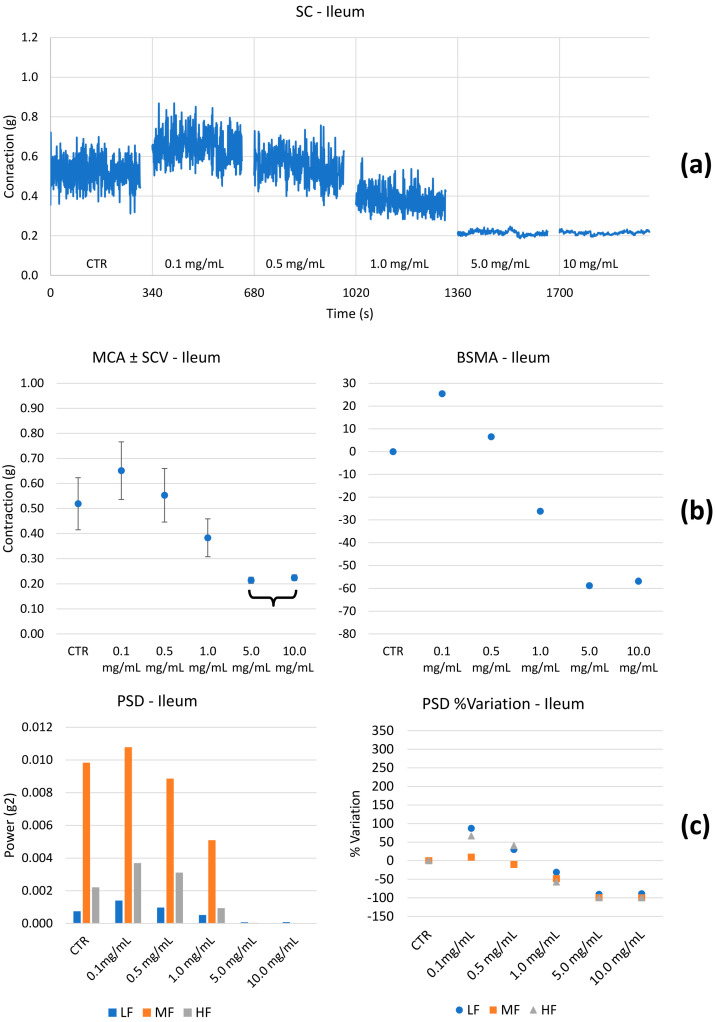
Example of experimental original recording of the concentration–response curve of HEMEO on spontaneous longitudinal (Long) ileum basal contractility. (**a**) Spontaneous contraction (SC) signals for each concentration; (**b**) mean contraction amplitude (MCA) and spontaneous contraction variability (SCV), represented as error bars in the MCA plot and contraction percentage variation for the control (BSMA) for each considered condition; not statistically significant differences (*p* > 0.05) between MCAs at different concentrations are reported in the graph with a curly bracket. All the comparisons not reported are to be considered significant (*p* < 0.05); (**c**) power spectral density (PSD) and percentage variations. Longitudinal tones present a biphasic behavior: it increases for 0.1 mg/mL concentrations and then decreases until a maximum difference of −60%. LF and HF present the same tend.

**Figure 5:**
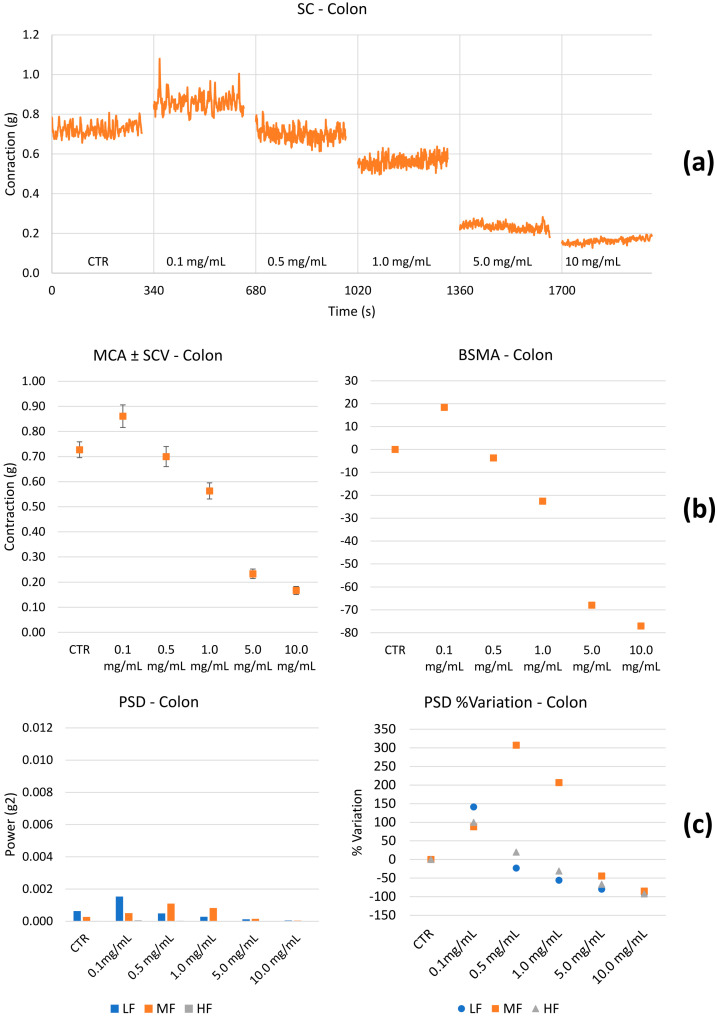
Example of experimental original recording of the concentration–response curve of HEMEO on spontaneous longitudinal (Long) colon basal contractility. (**a**) Spontaneous contraction (SC) signals for each concentration; (**b**) mean contraction amplitude (MCA) and spontaneous contraction variability (SCV), represented as error bars in the MCA plot and contraction percentage variation for the control (BSMA) for each considered condition; all the comparisons reported resulted statistically significant (*p* < 0.05); (**c**) power spectral density (PSD) and percentage variations. Longitudinal tones present a biphasic behavior: it increases for 0.1 mg/mL concentrations and then decreases until a maximum difference of −80%. LF and HF bands present the same biphasic trend. MF has a maximum increase of 300% at 0.5 mg/mL, then decreases after 5.0 mg/mL.

Spontaneous contractility. The effect of HEMEO on the spontaneous contractility of the gastric fundus, ileum, and colon were studied. Figure 3, Figure 4 and Figure 5 show the effects of HEMEO on the longitudinal stomach, ileum, and colon muscles’ spontaneous contractility.

Stomach: HEMEO effect on the longitudinal contractions of the stomach does not show physiologically significant effects on BSCA. The PSD percentage (%) variations for LF are not significant; differences in MF and HF contractions are significant only for the higher concentrations.

Based on our previous experience [60], LF related to the longitudinal smooth muscle is linked to transit speed: the higher the PSD band, the higher the transit speed. MF and HF are likely associated with pain. This last hypothesis was already proposed in the present study and verified for the ileum and colon; the same mechanism must be demonstrated for the stomach. Table 2 summarizes the results obtained; LF, MF, and HF were not significantly different in the stomach. Therefore, it can be argued that HEMEO does not modify tone and transit time, and its antibacterial activity could be carried out without the limit of accelerated emptying. The ileum and colon showed similar behavior. At low concentrations, transit velocity, pain, and tone increased. The parameters decreased for concentrations greater than or equal to 1.0 mg/mL (Tables S1–S6).

**Figure 6:**
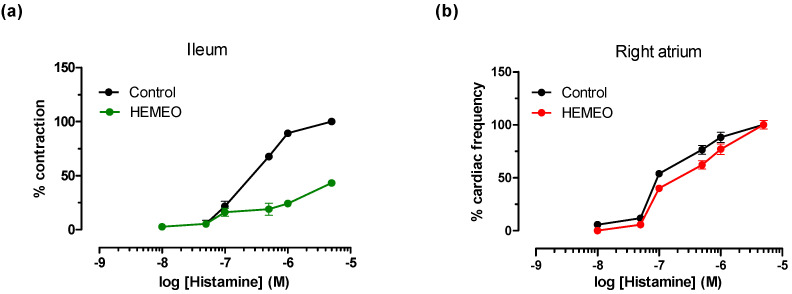
Effect of HEMEO on histamine-induced contraction in isolated guinea pig ileum (**a**) and in spontaneously beating right atrium (**b**). Cumulative concentration–response curves were obtained before and after exposure to HEMEO (1 mg/mL) for 30 min. Each point is the mean ± SEM (*n* = 5–6). Where error bars are not shown, these are covered by the point itself.

Histamine receptors. HEMEO was evaluated for its ability to modulate the activity of gut H_1_-histamine receptors. The H_1_ subtype in the gastrointestinal system contributes to the control of contractility [60], while the H_2_ subtype is prevalent in cardiac tissue, where it modulates heart rate [61]. Table 3 and Figure 6 show that HEMEO antagonizes histamine-induced contraction on the ileum in a non-competitive mechanism. These effects were reversible with 30 min of washing. The same study, conducted on the H_2_-histaminic receptor of cardiac tissue, gave a negative result: HEMEO (1 mg/mL) did not modify the increase in heart rate induced by histamine.

Calcium Channels. HEMEO effect on selected gastrointestinal tracts (gastric fundus, ileum, and colon) was evaluated to verify its potential spasmolytic effects on K^+^-induced contraction; the considered tracts had fundamental involvement in the digestive process. In addition, the effects of HEMEO were evaluated on guinea pig aorta smooth muscle. The obtained data are shown in Table 4. As can be seen, HEMEO did not inhibit potassium-induced stomach contraction; it showed a concentration-dependent spasmolytic action on the ileum and colon, with approximately four times greater potency on the ileum concerning the colon. In addition, the maximum concentration (10 mg/mL) on the aorta suggested a weak contractile effect.

Cardiac parameters. The cardiac profile of HEMEO was assessed at 1 mg/mL concentration on guinea pig isolated left and right atria, driven at 1 Hz to evaluate the inotropic effect. The chronotropic activity was also assessed by spontaneously beating the right atrium. The obtained data related to 1 h of incubation are shown in Table 5. HEMEO did not modify the force of cardiac contraction evaluated on the left atrium driven at 1 Hz and on the spontaneously beating right atrium, and it had no effects on the heart rate monitored on the right atrium in spontaneous activity.

### 3.4. Antioxidant Activity

**Figure 7:**
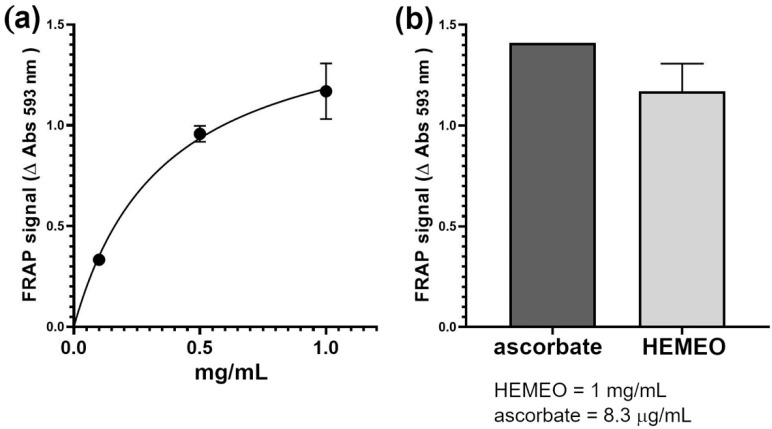
(**a**) The antioxidant power of the HEMEO solution was measured using the FRAP assay. (**b**) Comparison of 1 mg/mL HEMEO antioxidant power with the reference compound ascorbate.

The antioxidant capacity of HEMEO was evaluated using the FRAP assay, revealing a dose–response relationship with the different concentrations of HEMEO tested (Figure 7a). Specifically, a 1 mg/mL concentration of HEMEO generated a FRAP signal comparable to that produced by 8.3 µg/mL (equivalent to 47 nM) of ascorbate, used as a reference antioxidant compound (Figure 7b). The IC_50_ of HEMEO was estimated to be about 0.25 mg/mL).

### 3.5. Antimicrobial Activity

To assess the antimicrobial activity of HEMEO against *H. pylori*, a disk diffusion assay was performed using HEMEO (80 µg/disk) on Brucella Broth agar, incubated at 37 °C for 72 h. The mean zone of inhibition was 12 ± 2 mm, while negative control disks (solvent only) produced no inhibition, confirming compound activity (Table 6, Figure S4). The activity profile was similar to the reference antibiotic Kanamycin, even if the potency was considerably lower (Kanamycin, at a concentration of 2.5 mg/mL, inhibits growth by 35 ± 2). The results obtained in our assay align well with those reported for other extracts and preparations of similar complexity tested against *H. pylori* using the same methodology [62,63,64,65,66].

## 4. Discussion

HEMEO is an herbal mixture containing three essential oils formulated to harness the synergistic effects of various organic compounds that can simultaneously target multiple pathways associated with specific pathologies [15]. The native liquid state of the essential oils is suitable for most applications. However, the solid form is preferred when intended to be used as a component in oral dosage nutraceutical formulations. In HEMEO, conventional excipients such as silica were used to deliver the essential oils of *Foeniculum vulgare* Mill, *Mentha piperita* L., and *Pimpinella anisum* L. The results of the GC-MS analysis carried out on the formulation at different storage times demonstrated the stability of the HEMEO solid formulation.

By combining multiple herbs, HEMEO extends its therapeutic effects to a broader spectrum of conditions than it would with a single herb. Lowering the individual herb concentrations required may also help reduce off-target effects, decreasing the likelihood of unintended interactions with proteins or other molecular targets beyond the intended scope [58].

The chemical profile of HEMEO guided this study’s focus on its potential application in managing gastrointestinal contractility disorders, an essential aspect of many gastrointestinal conditions [67].

**Figure 8:**
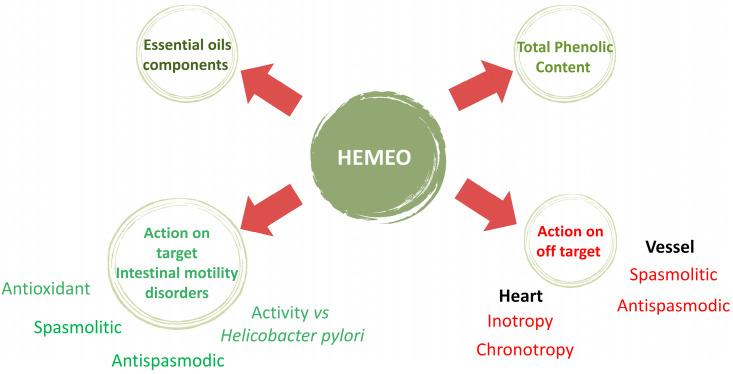
Schematic diagram showing the detail of the experimental design.

Peristaltic waves typically propel chyme along short segments of the intestine rather than the entire tract. However, the regulation of intestinal propulsion relies on the brain–gut axis, which is influenced by both extrinsic innervation (splanchnic and vagal–sacral pathways) and intrinsic networks (the myenteric and *submucosal plexi*). Much of the knowledge about gastrointestinal contractility comes from studies focused on specific GI regions or animal models, and these indicate that motility regulation mechanisms vary by anatomical site and muscle type. The expression of receptors, mechanisms for smooth muscle excitability, and regulation of contractile force all contribute to a highly complex brain–gut interaction system. A further challenge is that motility disorders may be localized, manifesting in specific gastrointestinal tract segments but not others. For example, hypomotility in the colon may lead to constipation without significantly affecting other segments. As a result, treatments aimed at increasing motility in one area risk inadvertently disrupting motility elsewhere [68]. A schematic diagram showing the detailed mechanism by which polyherbal extract protects against intestinal motility disorders is illustrated in Figure 8.

To evaluate the HEMEO activity, three key digestive processes—the stomach, ileum, and colon—were examined, assessing the effects on spontaneous and induced contractility and the outer layers of the guinea pig gut muscular tissues. The guinea pig is a well-known model for human intestinal studies and microbiome research and is predictive of translation to humans [69].

The latter contributes to contracting, relaxing, shortening, and lengthening the gut, providing the forces necessary to stir the gut contents and move food, water, and waste through the tubular chambers in one direction. The contractility of the longitudinal muscle is illustrated in the baseline of Figure 3, Figure 4 and Figure 5. The propulsion of the ingesta farther down the gastrointestinal tract is contributed by the segmenting contractions of the outer longitudinal muscle. HEMEO has been shown to not significantly affect the spontaneous contractility of the stomach at the low doses studied.

It is known that decreasing gastric smooth muscle tone may decrease symptoms induced by gastric filling in patients with gastric accommodation, improving functional dyspeptic symptoms due to the reduction in gastric tone and increased compliance following ingestion of a meal. Transit speed, pain, and tone were increased in the ileum at low doses (HEMEO at 0.1–0.5 mg/mL), but no effect was observed at high doses. In the colon, tone, transit speed, and pain increased at the lowest dose but remained unchanged or decreased at higher doses. The decrease in transit velocity suggests a longer residence time of the intestinal contents and, therefore, better mixing/absorption/digestion/enzymatic work in the ileum. The two maximal doses of HEMEO were significantly different from the base, and a biphasic trend was observed: at low doses, MCA and SCV increased, whereas, at the higher doses, the observed values were lower than the control. The decrease in tone is associated with a relaxation of the smooth muscle fibers, leading to a global improvement in the digestive process and less pain in the colon. For example, IBS, a well-known intestinal disorder, is associated with diverse pathophysiological mechanisms, including increased abnormal colonic motility or transit. HEMEO, at the high doses applied in the present study, was found to decrease pain, tone, and transit, suggesting that it could serve as a supportive treatment for increased abnormal colonic motility or transit and relieve pain in IBS (Table 2).

The biphasic effect observed with HEMEO may be attributed to the involvement of different receptor populations, as described in the literature for conventional drugs [70]. In the case of HEMEO, a blend of herbal extracts and essential oils, effects on multiple targets are highly plausible.

Regarding its effect on calcium movements in the gastrointestinal tract, HEMEO has shown a spasmolytic effect on potassium-induced contractions [71]. While 1,4-Dihydropyridines primarily target vascular muscles, thus limiting their use in intestinal motility control [72], otilonium bromide selectively affects intestinal smooth muscles [73]. Therefore, it proves helpful in inflammatory intestinal diseases and cannot affect heart parameters [49,74,75]. HEMEO selectively affects the ileum and colon without interfering with stomach contractility, and its lack of effect on vascular smooth muscle positions it as an off-target therapeutic option.

These results support HEMEO’s potential use in managing stomach atony. At the appropriate dose, HEMEO (Table 6) can control the growth of *H. pylori* while supporting healthy digestive function. *H. pylori* contributes to digestive disruptions. Once thought to be almost sterile due to its high acidity, the stomach hosts microorganisms, primarily *Firmicutes* [76]. In addition, proteolytic enzymes transform macronutrients into more easily absorbable molecules, proving to be an effective barrier against infections [77].

However, the environment allows for the growth of *H. pylori* microorganisms that can live and replicate at low pH values. In turn, *H. pylori* implements a series of mechanisms to modify the pH, making the surrounding environment alkaline and creating favorable conditions for its survival. *H. pylori* is primarily implicated in gastric diseases but has also been associated with various systemic conditions [11]. *H. pylori* infection triggers a series of events, including inflammation and reduction in secretory capacity, which leads to a clinical picture called atrophic gastritis [78]. *H. pylori* is also implicated in the onset of peptic ulcers by producing particular toxins that cause damage to the gastric epithelium associated with gastric diseases and cancer [79].

Clinical investigations have shown that *H. pylori* infection and the concomitant use of NSAIDs or acetylsalicylic acid increase the risk of peptic ulcer compared to patients who were proven to be susceptible to the infection [80].

All these insults produced by *H pylori* on the gastric wall highlight its essential role in the cascade leading to cancer development [81]. Conventional therapy involves using a combination of antibiotics and proton pump inhibitors [82]. The resistance to antibiotics induced by the continual use of conventional therapy has driven interest in the search for natural alternatives that can contribute to the control of this microorganism, suggesting the use of foods or plant extracts with proven action against *H pylori*, acting mainly through the inhibition of specific enzymatic pathways [83]. Among these, green tea chains and polyphenols such as the gingerols of *Zingiber officinalis* and essential oils such as those obtained from peppermint [84] have aroused interest.

HEMEO contains peppermint essential oils whose volatile compounds are known for their antibacterial action against this microorganism, contributing to the action of HEMEO on the *H. pylori* target [85]. The essential oil of *Pimpinella anisum,* rich in anethole, inhibits the growth of *H. Pylori* while simultaneously inhibiting the expression of COX-2 [86]. The literature describes the positive effects of combining *Glycyrrhiza glabra* with antibiotics [87]. At a concentration of 20 mg/mL, HEMEO inhibits the growth of *H pylori*, contributing to the maintenance of gastric function and exercising a protective role. In this case, the presence of *Glycyrrhiza glabra* increases the effectiveness of the mix. Still, the simultaneous presence of other extracts allows for the reduction in the concentration of *Glycyrrhiza glabra* phytocoplex, avoiding the known effects on the cardiovascular system, which represents an off-target effect [88]. Moreover, glycyrrhizin, a component of *Glycyrrhiza glabra*, has been shown to promote gastric mucosal healing, while glycyrrhizic acid exhibits spasmolytic effects, helping to alleviate gastrointestinal discomfort [89,90]. In our study, HEMEO exhibited a mild contractile impact, reaching a maximum of 12% at a concentration of 10 mg/mL, selectively acting on smooth muscles of the ileum and colon without compromising cardiac function. This selective action may provide a therapeutic advantage by targeting gastrointestinal motility without triggering systemic cardiovascular effects, which is significant given the cardiovascular side effects often associated with *Glycyrrhiza glabra* at higher concentrations. Moreover, other plant species, such as *Tabebuia*, have demonstrated anti-*H. pylori* properties [81].

Scientific literature has demonstrated that patients with *H. pylori* infection who are treated with L-type calcium channel blockers have a lower risk of developing gastric cancer [91]. HEMEO interferes with calcium movements similarly to well-known calcium modulators in cardiovascular [72] and gastrointestinal disorders [49,92].

In addition to its role in calcium modulation, HEMEO provides gastrointestinal support by incorporating *Asparagus racemosus* and *Foeniculum vulgare* essential oils, each contributing specific properties relevant to gastrointestinal health. *Asparagus racemosus* Willd. Oberm, an herbaceous plant from the *Lamiaceae* family, is predominantly used as a urinary antiseptic due to its methyl mercaptan content [93]. In Ayurvedic medicine, the roots are considered stomachic, and the extract is utilized in preparations for peptic ulcers [22], including those induced by prolonged ranitidine use [24], owing to its antimicrobial [23] and antifungal properties [94]. Studies by Bhatnagar et al. demonstrate that using *Asparagus racemosus* is associated with significant antidiarrheal activity by inhibiting intestinal motility [27]. Additionally, *Asparagus* is known for its detoxifying properties: Ayurvedic medicine traditionally uses this herb to purify the body and enhance overall health. It supports the elimination of toxins and promotes kidney function, thereby contributing to the body’s natural detoxification processes [95,96].

*Foeniculum vulgare* Mill, commonly known as fennel, is used in traditional medicine to treat various conditions, particularly digestion and respiration [34]. Traditional practices emphasize its antimicrobial and antifungal activities, confirmed for both aqueous extracts of aerial parts [35] and essential oil [36]. The essential oil from the seeds also exhibits hepatoprotective properties, likely due to *d-limonene* and *β*-*myrcene* [37]. It contributes to regulating intestinal motility and relieving gastrointestinal atony or discomfort [97]. Additionally, anethole, an essential compound in fennel, has shown anticancer properties [98]. *Tabebuia avellanedae* is traditionally recognized for its detoxifying effects, particularly its ability to enhance the body’s ability to eliminate toxins. Its compounds support liver function and promote overall health by purifying the blood and improving circulation. Recent studies highlight its antioxidant properties, contributing to immune system support and general well-being [99].

Histamine is present in the gastrointestinal system, especially during inflammatory processes. It is essential in numerous gastrointestinal pathologies, such as IBS. HEMEO modulates the histamine H_1_ receptor in a reversible, non-competitive manner in the intestine without altering the heart rate (Table 3, Figure 6). This result is particularly interesting, as it allows for the modulation of intestinal contractility without off-target effects. Given that HEMEO is a blend of multiple extracts, the observed TPC likely reflects an additive or synergistic effect, resulting in a level slightly above that of individual plant extracts. This increased TPC may enhance the overall antioxidant potential of HEMEO compared to its components, suggesting a comprehensive approach to mitigating oxidative stress, which is a disrupted parameter in most diseases.

The TPC of HEMEO, measured at 9.925 mg GAE/g, and its antioxidant capacity, comparable to 8.3 µg/mL of ascorbic acid, place it within a favorable range for natural extracts and substantial for multi-component blends. This observation aligns with research on various Mediterranean plants, highlighting a solid relationship between TPC and antioxidant efficacy [100]. It indicates that even moderate levels of polyphenols can yield significant antioxidative effects.

A significant limitation of this study is the small sample size analyzed. Our primary focus was the methodological approach to accurately assess the biochemical, bacteriological, antioxidant, and motility-related aspects of gastric and intestinal tissues exposed to drug effects. Further investigations are required to delve deeper into the toxicological properties of herbal preparations [101,102] and the essential oils in HEMEO [103] to validate our findings and evaluate inter- and intra-individual variability.

## 5. Conclusions

The diverse range of activities demonstrated by HEMEO highlights its potential application in the formulation of dietary supplements targeting various health needs. Its selective effect on ileum and colon motility, as defined by the Rome Foundation criteria, suggests potential benefits in managing functional motility disorders associated with multiple pathologies [104]. Additionally, the mixed herbal extract, including essential oils, inhibits the growth of *Helicobacter pylori* without impairing spontaneous or induced stomach contractions.

The presence of specific bioactive compounds—such as saponins in *Asparagus racemosus*, flavonoids and glycyrrhizin in *Glycyrrhiza glabra*, menthol in *Mentha piperita*, and anethole in *Pimpinella anisum*—is central to HEMEO’s therapeutic effects. These compounds, known for their anti-inflammatory, spasmolytic, antimicrobial, and antioxidant properties, significantly contribute to HEMEO’s efficacy in disease prevention and as an adjunct in managing specific gastrointestinal conditions.

In summary, HEMEO represents a promising candidate for further research and development in dietary supplements. Its multifaceted properties offer notable therapeutic advantages [105].

Nevertheless, although extensive information exists on the effects of individual components, further in-depth studies are ongoing to evaluate HEMEO’s effects in a translational context, particularly regarding its potential synergistic interactions with drugs [106].

## Figures and Tables

**Table 1:** HEMEO total phenolic content (TPC).

Concentration	TPC ^a^
HEMEO	9.925 ± 0.42

^a^ Total phenolic content, expressed as mg gallic acid equivalent GAE/g (*n* = 5) HEMEO.

**Table 2:** Effects on stomach, ileum, and colon of HEMEO, including transit speed, pain, and muscle tones.

	Stomach					Ileum					Colon				
mg/mL	0.1	0.5	1.0	5.0	10.0	0.1	0.5	1.0	5.0	10.0	0.1	0.5	1.0	5.0	10.0
Transit speed variation%	=	-	-	=	=	+	=	=	-	-	+	=	-	-	-
Pain%	=	=	=	+	+	+	=	-	-	-	+	=	=	-	-
Longitudinal contraction variation%	=	=	=	=	=	++	+	--	---	---	+	-	--	----	----

Percentage variations in transit speed and pain: “---”: ≤−500%; “--”: [−500%, −300%]; “-”: [−300%, −50%]; “=”: [−50%, +50%]; “+”: [+50%, +300%]; “++”: [+300%, +500%]; Percentage contraction variations: “----”: [−80%, −60%]; “---”: [−60%, −40%]; “--”: [−40%, −20%]; “-”: [−20%, 0%]; “=”: Not significant; “+”: [0%, +20%]; “++”: [+20%, +40%].

**Table 3:** Histamine receptor activity of HEMEO.

Ileum ^a^		Right Atrium ^b^	
**IC_50_ ^c^**	95% Conf-Lim.	IC_50_ ^c^	95% Conf-Lim.
0.78	0.66–0.87	#	

^a^ Guinea pig ileum smooth muscle. ^b^ Guinea pig spontaneously beating right atrium ^c^ The inhibition concentration (IC_50_) was expressed as mg/mL concentration and was calculated from concentration–response curves (Probit analysis by Litchfield and Wilcoxon with *n* = 5–6) [51]. # inactive up to 5 mg/mL.

**Table 4:** Spasmolytic effect of HEMEO on smooth muscle preparations.

Tissue	IA ^a^	IC_50_ ^b^	95% Conf-Lim.
Gastric Fundus	24.0 ± 2.6		
Ileum	63.0 ± 3.7	1.33	1.11–1.60
Colon	98.0 ± 1.3	5.31	4.84–5.84
Aorta	12.0 ± 0.3 ^c^		

^a^ Intrinsic activity (IA) expressed as percent inhibition of calcium-induced contraction on K^+^-depolarized (80 mM) guinea pig gastric fundus, ileum, colon, and aortic strips at 10 mg/mL. Data are presented as M ± S.E.M. ^b^ The 50% of the effect of concentration (IC_50_) was expressed as mg/mL and calculated from concentration–response curves (Probit analysis by Litchfield and Wilcoxon [51] with *n* = 6–7). When the maximum effect was <50%, the IC_50_ values were not calculated. ^c^ positive effect.

**Table 5:** Cardiac activity of HEMEO.

Left Atrium	Right Atrium	
Negative Inotropy ^a^	Negative Inotropy ^b^	Negative Chronotropy
IA ^a^	IA ^b^	IA ^c^
*	*	*

^a,b^ IA: intrinsic activity expressed as a decrease in developed tension on isolated guinea pig left atrium driven at 1 Hz and on guinea pig spontaneously beating right atrium at 1 mg/mL HEMEO concentration, expressed as percent change from the control (*n* = 5–6). ^c^ Decrease in the atrial rate of HEMEO (1 mg/mL) on guinea pig spontaneously beating right atrium. Pretreatment heart rate ranged from 165 to 190 beats/min. * inactive.

**Table 6:** HEMEO and positive control vs. *H. pylori* G27.

Sample	Concentration			
	2.5 mg/mL ^a^	20.0 mg/mL ^a^	100 mg/mL ^a^	MAQ ^b^
HEMEO	NT	12 ± 2	22 ± 4	20.0
Karnamicyn	35 ± 2	NT	NT	

^a^ Mean diameter of inhibition (mm ± SD). ^b^ MAQ (minimum active quantity) values expressed as mg/mL of samples tested for activity against *H. pylori* G27.

## Data Availability

Data are available upon request to the corresponding author.

## Supplementary Materials

The supporting files can be downloaded at: https://www.mdpi.com/article/10.3390/nu16244357/s1, Figure S1: Total ion current GC chromatograms of a standard solution of trans-anethole (a) and dichloromethane extract from the real sample (b) and their on-line mass spectra: trans-anethole standard (c) and real sample (d). Chromatographic and detection conditions are reported in the text; Figure S2: Total ion current GC chromatograms of a standard solution of estragole (a) and dichloromethane extract from the real sample (b) and their on-line mass spectra: estragole standard (c) and real sample (d). Chromatographic and detection conditions are reported in the text; Figure S3: Total ion current GC chromatograms of a standard solution of menthol (a) and dichloromethane extract from the real sample (b) and their on-line mass spectra: menthol standard (c) and real sample (d). Chromatographic and detection conditions are reported in the text; Figure S4: Zone of inhibition; Table S1: Spontaneous motility data of stomach tissue; Table S2: Effect size (ANOVA with Bonferroni’s correction) for stomach tissue; Table S3: Spontaneous motility data of ileum tissue; Table S4: Effect size (ANOVA with Bonferroni’s correction) for ileum tissue; Table S5: Spontaneous motility data of colon tissue; Table S6: Effect size (ANOVA with Bonferroni’s correction) for colon tissue.
